# Deep-Learning-Based Analysis Reveals a Social Behavior Deficit in Mice Exposed Prenatally to Nicotine

**DOI:** 10.3390/cells13030275

**Published:** 2024-02-01

**Authors:** Mengyun Zhou, Wen Qiu, Nobuhiko Ohashi, Lihao Sun, Marie-Louis Wronski, Emi Kouyama-Suzuki, Yoshinori Shirai, Toru Yanagawa, Takuma Mori, Katsuhiko Tabuchi

**Affiliations:** 1 Department of Molecular and Cellular Physiology, Shinshu University School of Medicine, Matsumoto 390-8621, Japan; 20hm124d@shinshu-u.ac.jp (M.Z.); qw66662024@126.com (W.Q.); nobuhiko24@shinshu-u.ac.jp (N.O.); 22hm124a@shinshu-u.ac.jp (L.S.); marie-louis.wronski@uniklinikum-dresden.de (M.-L.W.); emi_suzuki@shinshu-u.ac.jp (E.K.-S.); yoshirai@shinshu-u.ac.jp (Y.S.); 2 Neuroendocrine Unit, Department of Medicine, Massachusetts General Hospital and Harvard Medical School, Boston, MA 02114, USA; 3 Translational Developmental Neuroscience Section, Division of Psychological and Social Medicine and Developmental Neurosciences, Faculty of Medicine, TU Dresden, 01307 Dresden, Germany; 4 Department of Oral and Maxillofacial Surgery, Faculty of Medicine, University of Tsukuba, Tsukuba 305-8575, Japan; ytony@md.tsukuba.ac.jp; 5 Department of Neuroinnovation, Institute for Biomedical Sciences, Interdisciplinary Cluster for Cutting Edge Research, Shinshu University, Matsumoto 390-8621, Japan

**Keywords:** deep learning, prenatal nicotine exposure, ASD, ADHD, DeepLabCut, SimBA

## Abstract

Cigarette smoking during pregnancy is known to be associated with the incidence of attention-deficit/hyperactive disorder (ADHD). Recent developments in deep learning algorithms enable us to assess the behavioral phenotypes of animal models without cognitive bias during manual analysis. In this study, we established prenatal nicotine exposure (PNE) mice and evaluated their behavioral phenotypes using DeepLabCut and SimBA. We optimized the training parameters of DeepLabCut for pose estimation and succeeded in labeling a single-mouse or two-mouse model with high fidelity during free-moving behavior. We applied the trained network to analyze the behavior of the mice and found that PNE mice exhibited impulsivity and a lessened working memory, which are characteristics of ADHD. PNE mice also showed elevated anxiety and deficits in social interaction, reminiscent of autism spectrum disorder (ASD). We further examined PNE mice by evaluating adult neurogenesis in the hippocampus, which is a pathological hallmark of ASD, and demonstrated that newborn neurons were decreased, specifically in the ventral part of the hippocampus, which is reported to be related to emotional and social behaviors. These results support the hypothesis that PNE is a risk factor for comorbidity with ADHD and ASD in mice.

## 1. Introduction

Cigarette smoking is known to be a risk factor for many diseases, including cancer, heart disease, stroke, and diabetes, and has been shown to significantly increase the risk of some mental health disorders such as major depression [1]. Some studies have also found that cigarette smoking affects the embryonic development of humans, leading to failed delivery, high infant mortality, and decreased infant body weight [2,3,4,5]. Recently, more attention has been attracted to the hypothesis that cigarette smoking during pregnancy is associated with an elevated risk for neurodevelopmental disorders, such as attention-deficit/hyperactivity disorder (ADHD) [6,7,8,9] and autism spectrum disorder (ASD) [10,11,12]. Tobacco contains nicotine, which, via the mother’s blood, crosses the placenta and is effectively transferred to the fetus [13,14].

Nicotine is a nonselective agonist to nicotinic acetylcholine receptors (AChRs), which are distributed in the entire brain [15,16]. In the mammalian brain, twelve distinct subunits of nicotinic AChRs (nAChRs) have been identified, α2 to α10 and β2 to β4. Among the homomeric and heteromeric nAChRs, α4β2 nAChRs are the most common and account for more than 90% of nAChRs. Another one, α7 nAChR, is also widely distributed throughout the brain early in embryonic development. Among the various behavioral deficits associated with maternal cigarette smoking, its relationship with ADHD has been well studied in humans [9]. Rodent models of ADHD induced by maternal cigarette smoking have been established by the application of nicotine to pregnant animals [17,18,19,20,21]. Prenatal nicotine exposure (PNE) has been shown to cause behavioral changes in rodents, but the phenotypes are variable between reports. Some studies have reported anxiety-like responses, hyperactivity, and impulsivity, but other studies did not find a significant effect on behavior [22,23]. This contradiction may result from the difference among the PNE protocols and/or strains of animals.

In addition to the difference in the protocols used in the generation of model animals, the assessment of animal behaviors could be another reason for the inconsistency produced in these results. Until recently, the assessment of animal behaviors has relied on the efforts of human observers, which potentially includes the risk of psychologically biased analysis. The recent development of machine learning algorithms to analyze videos of animal behaviors allows us to identify the postures of animals with high accuracy, and open-source toolkits such as DeepLabCut [24] and SLEAP [25] have become widely available. These tools can label the body parts of animals with a markless video and precisely estimate their poses using supervised machine learning. Since animal behaviors are considered as the coordination of body parts in a short period, other toolkits based on deep learning algorithms use the pose estimation obtained with DeepLabCut and classify types of animal behaviors. Simple Behavioral Analysis (SimBA) is one of the open-source toolkits used to classify animal behaviors using supervised machine learning [26].

One of the technical difficulties in using these toolkits for analysis lies in attempting to achieve high accuracy in labeling the animals. Highly accurate labeling is quite challenging in the case of multiple animals because human experimenters must provide precise training data. We might use two different strains of animals with different fur colors, enabling us to discriminate between the individuals and label their body parts [24,26,27]. However, animal strains are known to exhibit different characteristic social behaviors, and it is recommended that researchers use the same strain to analyze social interactions and evaluate the sociability of animal models [28,29].

Various rodent models of ASD have been established by the introduction of gene mutations [30,31] or the application of chemicals such as valproic acid [32,33]. These models have been investigated using not only behavioral assays but also other biological methods, such as a histological analysis of the patterns of adult neurogenesis [34,35,36]. Even though clinical reports have indicated an association between smoking during pregnancy and the ASD child phenotype, the behavioral characteristics of PNE animals have been unclear and contradictory.

In this current study, we applied nicotine to pregnant C57BL/6J mice and examined the behavioral characteristics of the mice (PNE mice) using automated analysis and studied the adult neurogenesis patterns in the hippocampus. We produced a PNE mouse model with the behavioral features of ADHD, which were confirmed by both human experimenters and a machine learning algorithm. We then optimized a network of DeepLabCut to label body parts of two C57BL/6J mice with the same fur color with high accuracy. The precise labeling of the body parts enabled us to analyze the social interaction between the PNE mice and the juvenile target mice, which was confirmed by a traditional assessment by human experimenters. PNE mice exhibited deficits in social interactions, indicating that PNE mice have an aspect of ASD. Our results show that comorbid ADHD and ASD are induced by prenatal exposure to nicotine in mice.

## 2. Materials and Methods

### 2.1. Animal Care and Administration of Nicotine to Pregnant Mice

All procedures of the animal experiments were reviewed by the Committee for Animal Experiments of Shinshu University and approved by the president of Shinshu University. Mice were group-housed under environmentally controlled conditions (12:12 light/dark cycle, 22 ± 2 °C, and 55 ± 10% relative humidity) with food and water ad libitum. Pregnant female C57BL/J mice were purchased from Japan SLC. They had free access to food and water. Water containing nicotine (200 µg/mL) and 2% sucrose (PNE group) or only 2% sucrose (control group) was prepared from gestational day 14 (G14) [20]. The postnatal day (P0) was defined on the day of the delivery, and pups were weighed at P1, 3, 5, 7, 10, 14, 21, 28, and 35 [37]. Eight PNE and eight control mice (from three pregnant mothers for both groups) were analyzed for developmental changes in body weight. Twenty-four PNE (from six mothers) and twenty-four control (from six mothers) mice were used to calculate the probability of survival. Four-week-old male mice were weaned and housed in a group of brothers until the day of the experiments.

### 2.2. Animal Behavioral Tests

#### 2.2.1. Habituation

Mice were housed individually 7 days before the day of the behavior test. We used a clear Plexiglas tube (with a diameter of 50 mm and length of 200 mm) for the tunnel handling to habituate animals. The mice stayed in the home cage before and after the behavioral tests.

#### 2.2.2. Cliff Avoidance Reaction (CAR) Test

We used an open-field arena (W40 cm × D40 cm × H40 cm) made of an opaque grey Plexiglas plate. A transparent Plexiglas cylinder with a diameter of 12.5 cm and a height of 25 cm was placed at the center of the open-field arena. The top of the cylinder was covered with a round plate of white-matt polyethylene to reduce the reflection of the room light [38]. A subject mouse was gently placed on the platform with the habituation tube, and its behavior was recorded for 15 min. If a mouse jumped off, it was returned to the platform. The elapsed time until a mouse first jumped off and the number of jumps were measured. In the case where a mouse did not jump off, the time was measured as 900 s. The number of jumps and the time were measured by experimenters who were blind to the conditions of the test mouse. We labeled the parts of the mouse body with DeepLabCut (as described below). The center of mass of seven key labeling points was used to draw the trajectory of the mouse during the experiment. Eleven PNE male mice from five pregnant mothers and eleven control male mice from four pregnant mothers were examined in this test.

#### 2.2.3. Y-Maze Test

The Y-maze arena consisted of three white opaque plastic arms (35 cm length, 6 cm width, and 20 cm height) at a 120° angle from each other [39]. A mouse was introduced in one of the arms and was allowed to explore freely for 8 min. Arm entry was defined when all four limbs of the mouse were within the arm, and spontaneous alternation was defined as the entry to the arm that was not used in the previous two entries. The number of arm entries was counted by an experimenter who was blind to the background of the test mouse. The alternation percentage was calculated to divide the number of spontaneous entries with the total number of entries to an arm. Ten PNE male mice from three pregnant mothers and ten control male mice from three pregnant mothers were examined in the test.

#### 2.2.4. Open-Field Test

An open-field test was performed as described previously [31]. A mouse was placed at the corner of the open-field arena and allowed to freely explore the chamber for 30 min. The behavior of the mouse was captured from the top of the open-field arena using a GoPro 10 Black camera at a resolution of 1920 × 1080 pixels and a frame rate of 30 frames per second. Two experimenters (Ex1 and Ex 2) manually counted the frequency and duration of grooming and rearing behaviors in the video. The video was analyzed with DeepLabCut, and the seven key points of the mouse body were labeled. The position of the mouse on a single frame of the video was defined as the center of the mass of all seven key points. The travel distance was calculated to sum the distance of a mouse’s movement between the sequential frames. The center area was defined as the square (20 cm × 20 cm), which was 10 cm away from the walls of the open-field arena. The time spent in the center square was measured. Twelve PNE male mice from five pregnant mothers and twelve control male mice from five pregnant mothers were examined in the open-field test.

#### 2.2.5. Juvenile Interaction Test

A previous protocol was modified and applied for the juvenile interaction test [40,41]. Five-week-old subject (Con and PNE) and three-week-old juvenile mice were used in the test. The juvenile mice were housed individually 1–3 days before the day of the experiment. On the day before the experiment, all mice were individually habituated to the test chamber for 10 min. The subject mouse was first placed on a corner of the open-field arena, and a juvenile mouse was introduced to the opposite corner of the arena. Both groups of mice were allowed to explore freely, and the videos were captured by a GoPro10 black for 10 min for further analysis. Human experimenters, who did not have any information regarding the background of the mouse, analyzed the frequency and the duration of sniffing and following behaviors. The travel distance and the time spent in the center were calculated as described in the open-field test. Twelve PNE male mice from five pregnant mothers and twelve control male mice from five pregnant mothers were in the juvenile interaction test.

### 2.3. Network Construction and Evaluation for Behavioral Analysis Using DeepLabCut

For the construction of networks for analyzing single mouse behavior and social interaction between two mice using DeepLabCut (DLC), 10 min videos were recorded in each scenario. All videos were precisely trimmed to 10 min, consisting of 18,000 frames, using Clipchamp v. 2.8.3.0, a free editing software. The male C57BL/J mice, aged 4–6 weeks, and the juvenile male mice, aged 3 weeks, involved in the interaction were obtained from SLC. A gray chamber (40 cm × 40 cm × 40 cm) was used for the test arena, which was illuminated by house lights with consistent white noise to obscure external sounds. DLC version 2.1.9 was installed on a Windows OS computer with an Intel Core i7-10700 2.9 GHz processor and an Nvidia GeForce RTX 3070 graphics card. The recorded videos were loaded into DLC, and a certain number of frames per video were randomly extracted. Subsequently, seven key points (nose, left ear, right ear, body center, left side, right side, and tail base) were manually labeled in each frame. The dataset was split into 95% for training and 5% for testing. The dlcnet_ms5 architecture was employed as the training model.

DLC calculated the root mean square error (RMSE) to quantify the discrepancy between the machine-labeled and human-labeled coordinates of the body parts. This metric allowed us to assess the accuracy of the model’s predictions. Moreover, when the trained model was applied to new videos, DLC provided x- and y- coordinates for each body part, along with a likelihood value ranging from 0 to 1.

To determine the optimal network configuration, we constructed various networks by altering the number of the videos (V), labeled frames per video (L), and iterations (I). The network configurations generated were as follows: 3V_20L_50000I, 3V_20L_200000I, 3V_20L_500000I, 3V_50L_200000I, 3V_100L_200000I, 5V_20L_200000I, and 10V_20L_200000I. Subsequently, we evaluated their performance based on RMSE values and examined the number of missing (NA) data points and data points with a likelihood value less than 1.0 using a custom R script. A comprehensive evaluation of these different networks was conducted on a separate set of 5 videos, and the obtained results were subjected to statistical analysis.

### 2.4. Supervised Behavioral Classification Using SimBA

SimBA was employed for behavior analysis within a dataset that comprised behavior videos and corresponding tracklet data generated by DeepLabCut (DLC). The process involved segmenting 15 videos from open-field testing to classify rearing and grooming, involving a total of 810,000 frames, and classifying following and sniffing in 15 videos from the juvenile interaction test, which encompassed 270,000 frames.

Each video was accompanied by predefined parameters, including frame rate (fps), resolution, and pixel measurements (px/mm). An outlier correction tool was employed to identify and correct pose estimation tracking inaccuracies by detecting outliers based on the movements and locations of the nose and tail base body parts in relation to the body lengths.

The video-specific parameters (pixels/mm and fps) were integrated with the corrected tracking data, enabling the calculation of a comprehensive set of variables, encompassing distances, movements, angles, and areas. Behavior annotations were manually generated to facilitate predictive classifier training in all curated videos, with one video set aside for validation purposes. The development of the behavior prediction model adhered to default training, hyperparameters, and evaluation settings.

All the resulting machine-generated data files were archived for subsequent analysis. To validate the accuracy of this deep-learning-based behavioral analysis approach, a careful comparison was conducted between the results generated by DLC plus SimBA and behavior assessments by multiple human annotators, which was considered the gold standard.

### 2.5. BrdU Injection and Histological Analysis

Bromodeoxyuridine (BrdU) was injected intraperitoneally (i.p.) at a dose of 150 mg/kg from the age of P42. Mice were injected three times daily for five consecutive days and survived for two weeks after the last injection of the BrdU. The mice were deeply anesthetized with a cocktail including medetomidine hydrochloride (Domitor, 0.3 mg/kg), midazolam (Dormicum, 4.0 mg/kg), and butorphanol tartrate (Vetorphale, 5.0 mg/kg). A total of sixteen brains (from eight PNE male mice delivered from two mothers and eight control male mice from two mothers) were analyzed in this study. All these mice were not examined in any behavioral tests.

Histological procedures were followed according to our previous protocols [42,43]. Mice were perfused with PBS and 4% paraformaldehyde in PBS two weeks after the last injection of BrdU. Subsequently, brains were decapitated, immersed in 4% PFA overnight, and soaked in 30% sucrose in PBS at 4 °C until they sank. Forty-micrometer-thick coronal sections were prepared from the brain using a freezing microtome. The brain sections were treated in PBS for 15 min and incubated in a 1 M hydrochloride solution for 30 min at 45 °C. After a 15 min wash in PBS at room temperature, the brain sections were incubated in the blocking buffer (PBS containing 2% normal donkey serum and 0.3% Triton X-100) for 1 h at room temperature. The brain sections were then incubated in the blocking buffer containing primary antibodies overnight at 4 °C. In this study, we used mouse anti-NeuN (MAB377, Roche, 1:1000 dilution) and rat anti-BrdU (ab6326, Abcam, 1:200) antibodies in the blocking solution as the primary antibodies. After 3 × 5 min washing with PBS, the brain sections were incubated in secondary antibodies, Alexa-488-conjugated donkey anti-mouse IgG and Alexa-594-conjugated donkey anti-rat IgG (both from Thermo, 1:400 dilution in PBS with 0.3% Triton X-100), for 2–3 h at room temperature. After the further washing of brain sections with PBS, they were mounted on glass slides, counterstained with DAPI, and coverslipped.

Images of coronal brain sections were obtained using a confocal microscope (SP8, Leica, Wetzlar, Germany) or a fluorescent microscope (BZ-X800, Keyence, Osaka, Japan) equipped with a 10× objective lens. Groups of images taken by BZ-hX800 were stitched using Image analyzer software v. 1.1.2.4 (Keyence). To estimate newborn neurons in different functional segments in the hippocampus, we divided the hippocampal formation into septal (dorsal) and temporal (ventral) regions. We first positioned the brain sections to a mouse brain atlas. Sections located before bregma −2.80 mm were categorized as dorsal dentate gyrus, while those after −2.80 mm were classified as ventral dentate gyrus in serial coronal brain sections. We then separately quantified newborn neurons in the dorsal and ventral segments. We selected one section for every sixth section and counted newborn neurons. Here, we defined newborn neurons as cells that were immunopositive for NeuN and BrdU in the dentate gyrus of the hippocampus. To estimate the total number of newborn neurons throughout a segmentation of the dentate gyrus, the average cell count (the total number of cells counted divided by the number of sections used for cell counting, approximately eight brain sections) was multiplied by the total number of sections with the hippocampal segmentations obtained from the animal (dorsal and ventral brain sections were approximately forty-five and thirty brain sections, respectively) [35]. The total number of newborn neurons in the whole dentate gyrus was calculated as the sum of the values for the dorsal and ventral segments.

### 2.6. Manual Analysis of Behaviors and Statistical Analyses

Human experimenters (Ex1 and Ex2) conducted manual analysis, and another experimenter labeled using DeepLabCut, and they shared behavior definitions prior to analysis. Ex1 and Ex2 were blind to the mice information when they analyzed the video. Another human experimenter also lacked information regarding the video and labeled parts of the mouse body on DeepLabCut and selected frames of mice behavior on SimBA.

All values were expressed as means ± SEM. Statistical significance was evaluated by using Student’s *t*-test for two groups or a Dunnett’s test for multiple groups. To compare the distribution of the interval distance between mice, the Kolmogorov–Smirnov test was applied to examine statistical significance. Statistical significance is indicated by asterisks (* *p* < 0.05, ** *p* < 0.01, *** *p* < 0.001). Circle markers on the bar graphs represent values obtained from individuals. Statistical analysis was carried out by Graphpad Prism 8 or R custom-made programs.

## 3. Results

### 3.1. Prenatal Nicotine Exposure Mice Exhibited Impulsive Behavior and a Deficit in Working Memory

**Figure 1:**
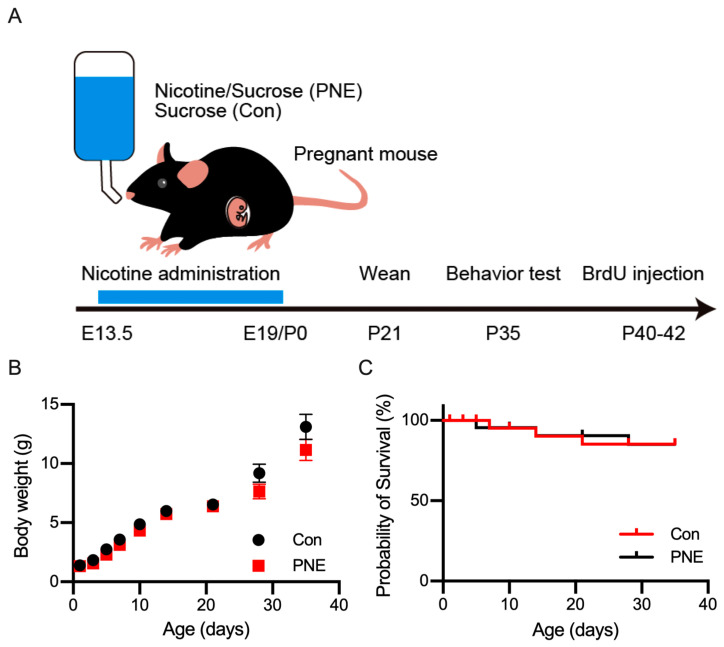
Establishment of nicotine exposure mice and postnatal development. (**A**): schematic drawing showing the experimental design of the prenatal nicotine exposure (PNE). Water with nicotine and sucrose (PNE), or sucrose only (Con), was supplied to pregnant C57BL/6J mice, starting from embryonic day 14 until delivery, postnatal day 0 (P0). Mothers and pups were supplied with normal drinking water thereafter. (**B**): the developmental changes in the body weight of PNE (*n* = 8) and Con (*n* = 8) pups until the date of the behavioral experiments. A slight decrease in the body weight was observed in PNE mice, but this was not statistically significant. (**C**): the survival curve of the PNE (*n* = 24) and Con (*n* = 24) mice indicated no change in the mortality after prenatal exposure of nicotine.

To generate PNE model mice, we followed a previously described protocol [20]. Water with 200 µg/mL of nicotine and 2% sucrose was supplied to pregnant females at E14 until the day of delivery (Figure 1A). Dams treated with nicotine during embryonic development (PNE) and treated with only 2% sucrose (Con, control) developed similarly toward adulthood (Figure 1B). Even though delivery failure occurred more frequently in the nicotine-treated pregnant females, the mortality of the pups was unaltered between the PNE and Con groups (Figure 1C).

**Figure 2:**
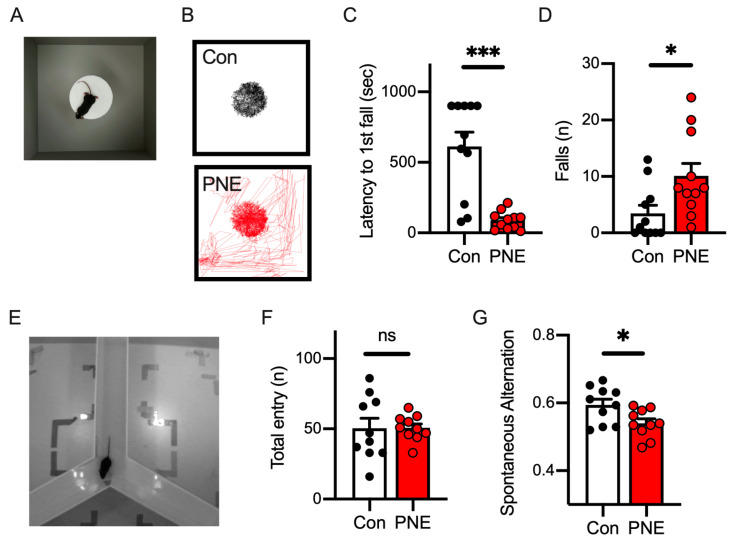
Nicotine exposure during the late embryonic stage demonstrated impulsivity and impaired working memory in mice. (**A**): the top view of the cliff avoidance reaction (CAR) test is shown. A test mouse was placed on a platform (20 cm high), and we observed whether it jumped from the platform. (**B**): the trajectory of the mice during the CAR test is shown. The Con mice stayed on the platform for the entire period of the CAR test (**top**), whereas the PNE mice jumped off the platform and walked on the floor (**bottom**). (**C**): the time elapsed to the first jump demonstrated that PNE mice (*n* = 11) jumped off the platform sooner after the CAR test started (t(20) = 5.07, *p* < 0.001). Some Con mice (*n* = 11) never jumped off from the platform. (**D**): the number of jumps during the 15 min CAR test showed that PNE mice jump from the platform more frequently than Con mice (t(20) = 2.515, *p* < 0.05). (**E**): the picture represents the top view of the Y-maze test. (**F**): the total arm entries are shown and indicate that the activity level was not altered by prenatal exposure to nicotine (PNE: n = 10, Con: n = 10, t(18) = 0.039, *p* > 0.1). (**G**): the percentage of spontaneous alternation was decreased in the PNE mice compared to the control mice (t(18) = 2.52, *p* < 0.05). Each bar represents the mean ± SEM; ns = not statistically significant, and * *p* < 0.05 and *** *p* < 0.001 was determined by using Student’s *t*-test.

Since prenatal nicotine exposure has been reported as a risk factor for ADHD both in humans and rodents [6,20], we applied behavioral tests to PNE mice to assess their behavioral characteristics. We first examined the impulsivity of the PNE mice using a modified cliff avoidance reaction (CAR) test. A mouse was placed on a platform of 20 cm height in an open-field arena; we observed PNE mice jump from the platform and walk on the floor of the arena, whereas Con mice sometimes stayed on the platform for the whole duration (Figure 2A,B). The time elapsed before the first jump was shorter in PNE mice than in the control mice (Figure 2C), and PNE mice jumped more frequently compared to the control mice (Figure 2D). These results indicate that exposure to nicotine in late embryonic development increases impulsivity in mice.

We next examined the working memory of PNE mice using the Y-maze spontaneous alternation test (Figure 2E), through which the working memory of the mice can be evaluated according to the percentage of arm entry alternation [39]. Even though the total number of arm entries did not alter between the control and PNE mice (Figure 2F), spontaneous arm entry alternation was higher in the control mice than in PNE mice (Figure 2G), suggesting that a deficit in working memory occurred in PNE mice.

### 3.2. Pose Estimation of a Single Mouse in the Open-Field Test by Using DeepLabCut

**Figure 3:**
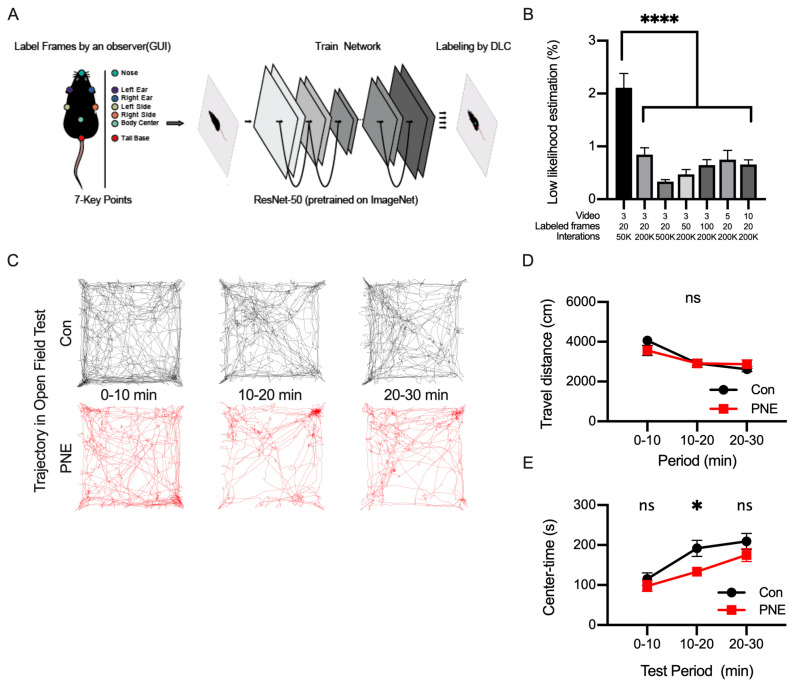
Labeling body parts of a single mouse using DeepLabCut and tracking the trajectory of the freely moving mouse. (**A**): DeepLabCut was used to label seven key points of the mouse during the open-field test, consisting of the nose (green), the left ear (purple), the right ear (blue), the left side of the trunk (grey), the right side of the trunk (orange), the body center (light green), and the base of the tail (red). These points were labeled by a human experimenter (training data) and the network was trained using the training data. (**B**): the graph shows the percentage of mislabeled frames in the video proceeded by different parameters to construct a network in DeepLabCut (F(6,28) = 16.09, *p* < 0.001). (**C**): the position of the mouse was estimated as the center of mass of the total parts of the mouse body labeled by DeepLabCut, and the trajectory of the mouse is shown. The trajectory of the control (black, n = 12) and PNE (red, n = 12) mice every 10 min is shown. (**D**): the travel distance of the control and PNE mice was not different in all the periods (0–10 min: t(22) = 1.44, *p* > 0.1; 10–20 min: t(22) = 0.03, *p* > 0.1; 20–30 min: t(22) = 1.01, *p* > 0.1). (**E**): the length of time during which the mice stayed in the center part of the open-field arena is shown. Control mice stayed in the center part longer during the 10-to-20 min period (0–10 min: t(22) = 0.85, *p* > 0.1; 10–20 min: t(22) = 2.61, *p* < 0.05; 20–30 min: t(22) = 1.34, *p* > 0.1), indicating that the control mice became accustomed sooner than PNE mice. ns = not statistically significant, * *p* < 0.05 and **** *p* < 0.0001 determined by using Student’s *t*-test.

We next examined the activity of the mice in the open-field test using a deep-learning-based method. We introduced a PNE or control mouse into the open-field arena and let the mouse explore freely for 30 min. We recorded a top-view video during the whole period of exploration. We imported the videos into DeepLabCut and labeled seven points on the mouse body (Figure 3A). Networks were constructed in DeepLabCut by changing the numbers of videos, the number of frames in each video, and the number of iterations during training. To evaluate the quality of pose estimation by the network, we calculated the percentage of frames with unavailable (NA) values or values lower than 1.0 (Figure 3B). We first evaluated the established network with three videos, twenty frames per video, and fifty thousand of iterations, of which approximately two percent of the frames were unlabeled. We then changed one or more training parameters and observed the increases in any of these parameters; the number of iterations is the most crucial factor affecting the quality of pose estimation in DeepLabCut. We also evaluated the RMSE metric, an index of the quality of network performance, and found that RMSE ranged from 0.15 to 0.21 cm in train error and from 0.17 to 0.26 cm in test error, respectively (Supplementary Figure S1). Based on these evaluations, we used the network established by the parameter set of 3 videos/20 frames/500 K iterations.

We used the mouse labels of the network on DeepLabCut and then examined the activity of the PNE and control mice in the open-field arena (Supplementary Video S1). The center of mass of the seven body points was used to define the location of the mouse, and the trajectory of the mouse over a 30 min period was analyzed (Figure 3C). We calculated the distances traveled every 10 min; this decreased toward the end of the open-field test and the distance did not alter between the PNE and control mice (Figure 3D). We also calculated the time spent in the center part of the arena as an index representing the anxiety level of the animal in a novel environment (Figure 3E). We found that the anxiety level of the control mice decreased 10 min after the open-field test, whereas PNE mice preferred to stay around the edge of the open-field arena.

### 3.3. Deep-Learning-Based Classification of Behaviors in a Single Animal by Using SimBA

**Figure 4:**
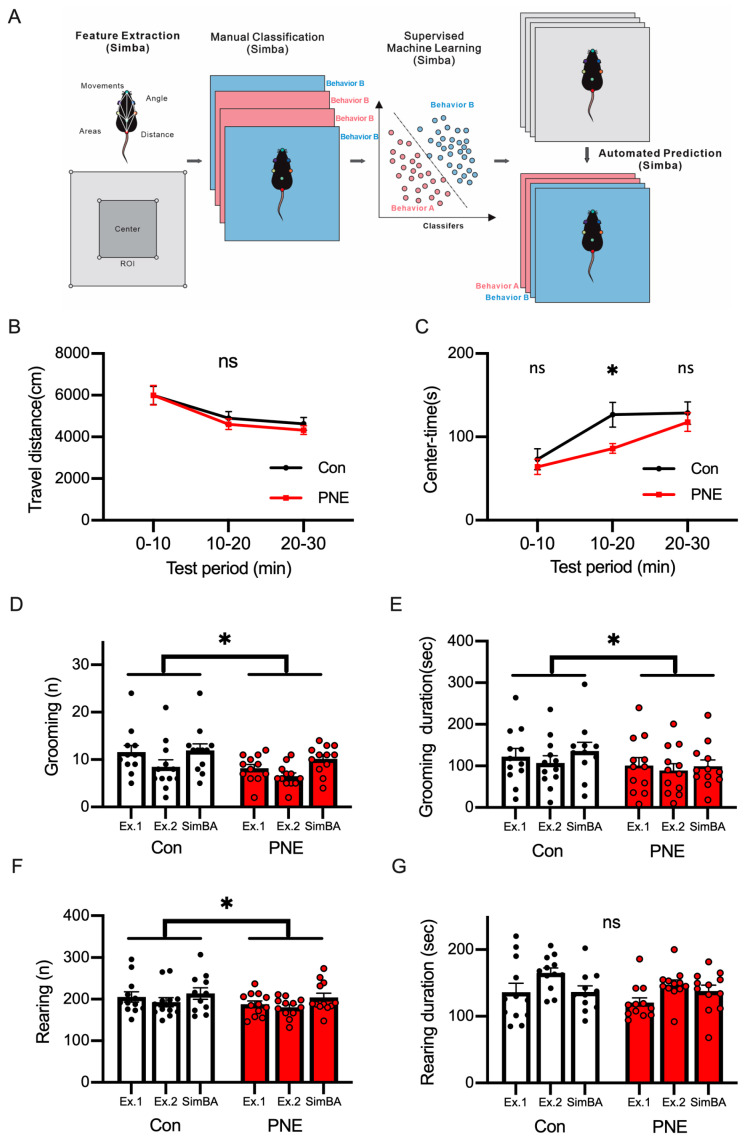
Machine-learning-based classification of mouse behaviors using SimBA achieved a detection of behaviors similar to human experimenters. (**A**): the scheme of the machine-learning-based classification of mouse behavior by using SimBA is shown. After the mouse pose estimation using DeepLabCut, pose features were extracted. A human experimenter defined behaviors in the video through manual classification on SimBA to define the training data. The training data were used to classify the mouse behaviors with supervised machine learning. The trained network was used to detect the behaviors of the mice in the video. We analyzed grooming (shown in (**D**,**E**)) and rearing (shown in (**F**,**G**)) behaviors in this study. (**B**): DLC/SimBA analysis did not detect significant changes in travel distance in each period (0–10 min: t(22) = 0.016, *p* > 0.1; 10–20 min: t(22) = 0.71, *p* > 0.1; 20–30 min: t(22) = 0.81, *p* > 0.1). (**C**): the decrease in the time spent in the center in PNE mice (n = 12) was detected by DLC/SimBA, in comparison with Con mice (n = 12; 0–10 min: t(22) = 0.58, *p* > 0.1; 10–20 min: t(22) = 2.55, *p* < 0.05; 20–30 min: t(22) = 0.62, *p* > 0.1). (**D**): the total frequency of grooming in the open-field test is shown and the frequency of grooming was decreased in PNE mice (t(2) = 4.60, *p* < 0.05). (**E**): the time spent grooming showed a decrease by PNE (t(2) = 4.48, *p* < 0.05). (**F**): the total frequency of rearing behaviors decreased in the PNE mice (t(2) = 5.85, *p* < 0.05). (**G**): the duration of rearing behavior did not change (t(2) = 1.75, *p* > 0.1). ns = not statistically significant, * *p* < 0.05, by using Student’s *t*-test.

We next used an open-source toolkit, Simple Behavioral Analysis (SimBA) to classify and annotate animal behaviors [26]. We obtained the pose estimation using DeepLabCut, as shown in Figure 3, extracted behavioral features on SimBA, and classified behaviors manually on SimBA (Figure 4A). After using supervised machine learning, we analyzed the videos through the classifier trained on SimBA and measured the distance traveled (Figure 4B) and the time spent in the center (Figure 4C). We used the SimBA network to detect grooming and rearing behaviors (Supplementary Video S2). To evaluate the quality of the behavioral classifier of SimBA, two experimenters analyzed the same set of videos to measure the frequencies and the durations of grooming and rearing behaviors (Figure 4D–G). The frequency (Figure 4D) and the duration (Figure 4E) of grooming decreased between the control and PNE mice. The frequency of rearing behavior was altered, but we did not detect any changes in the duration of rearing behavior between the PNE and control mice (Figure 4F,G). We examined the correlation between the observations by human experimenters and the detection using machine-learning-based analysis (Supplementary Figure S2) and found high correlations, indicating that the machine-learning-based analysis used in this study could replicate human analysis.

### 3.4. Pose Estimation of Two C57BL/6J Mice by Using DeepLabCut Requires Training Data from Diverse Videos and Frames

**Figure 5:**
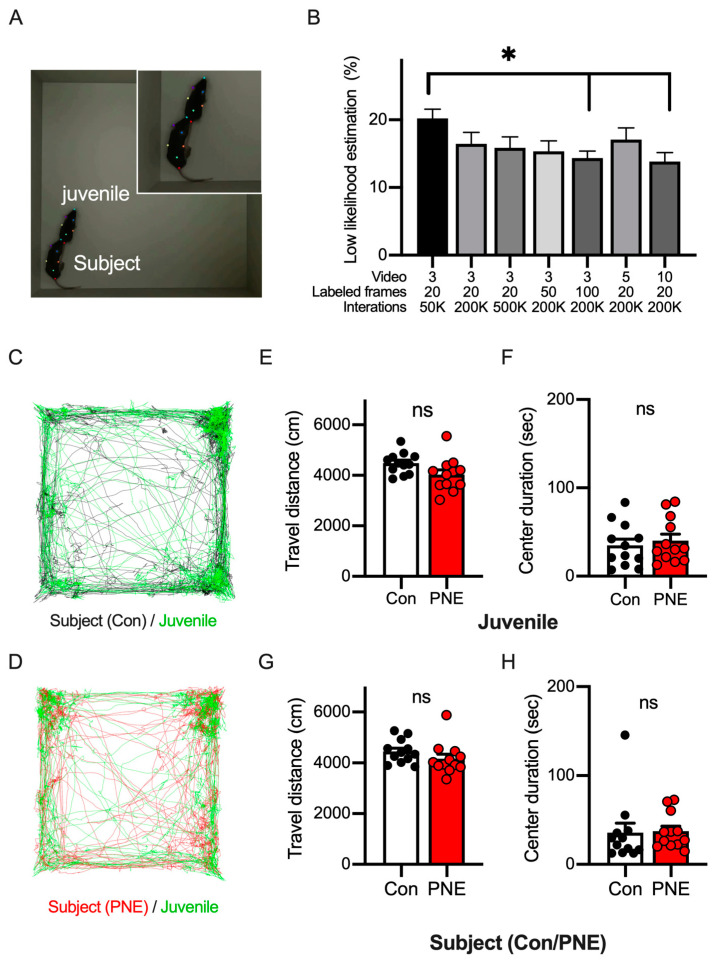
The pose estimation of two C57BL/6J mice in the open-field arena by using DeepLabCut allows us to track the trajectory of the mice. (**A**): the picture shows the top view of the social interaction test in the open-field arena with juvenile and older subject mice. (**B**): the graph shows the percentage of mislabeled frames in the video of the juvenile interaction test proceeded by different parameters to construct a network in DeepLabCut (F(6,28) = 1.998, *p* < 0.05). (**C**): the traces show the trajectory of the juvenile (green) and the control subject (black) mice in the open-field arena. (**D**): the traces show the trajectory of the juvenile (green) and the PNE subject (red) mice in the open-field arena. (**E**): the total travel distance of the juvenile mouse model is shown. The open bar represents the data when the juvenile mouse model was with the control subject mouse model (n = 12), and the red bar represents those with the PNE subject mouse model (open bar, *n* = 12, t(22) = 2.03, *p* = 0.06). (**F**): the duration of time the juvenile mice stayed in the center of the arena is shown (red bar, *n* = 12, t(22) = 0.52 *p* > 0.1). (**G**): the total distance of the subject mouse model is shown. The data of the control (open bar, *n* = 12) and PNE (red bar, *n* = 12) mouse models are shown (t(22) = 1.27, *p* > 0.1). (**H**): the duration of time the subject mouse model stayed in the center of the arena is shown (t(22) = 0.13, *p* > 0.1). Asterisk (* *p* < 0.05) was examined with ANOVA Turkey’s post hoc test. No statistical significance (ns, *p* > 0.05) was detected in the travel distance or the time spent in the center when using Student’s *t* test.

Smoking during pregnancy is known to be a risk factor for autism spectrum disorders, as well as ADHD. To investigate whether social behaviors may be affected by prenatal nicotine exposure, we examined the interaction between two freely moving mice in an arena (Figure 5A). We introduced a younger juvenile male mouse (3-week-old) into the open-field arena where an older subject mouse (5-week-old) explored and recorded a video for 10 min. This combination of ages of black mice allowed us to distinguish individual mice with the same fur color by body size and enabled us to generate training data in DeepLabCut or by following animals on the video manually. As previously described, we compared the training parameters to establish the best pose estimation by using DeepLabCut and found that high-quality pose estimation was achieved by a network trained using more videos (Figure 5B). The RMSE metric demonstrated excellent network performance in all sets of parameters, with the train error ranging from 0.16 cm to 0.21 cm and test error ranging from 0.20 cm to 0.28 cm (Supplementary Figure S3). Thus, we set the network model with 10 videos/20 frames/200 K iterations due to this setup having the highest accuracy in the pose estimation of multiple animals.

To assess the locomotor activity and anxiety levels of the PNE mice in the situation together with another mouse, we labeled the two mice (the subjects and the juveniles) in the open-field arena (Figure 5C,D) using the established network (Supplementary Video S3). The distance traveled by the juvenile mice was similar in both cases together with the PNE and control mice in the open-field area (Figure 5E), as was the time spent in the center part of the field (Figure 5F), indicating that PNE mice did not affect the activity and anxiety levels of the juvenile mice. We also investigated the distance traveled (Figure 5G) and the center-time (Figure 5H) of the subject mice, and neither of these parameters were altered between the control and PNE mice, suggesting that the juvenile mice did not affect these parameters in the subject mice.

### 3.5. Social Behavior Was Altered by Prenatal Exposure to Nicotine in Mice

**Figure 6:**
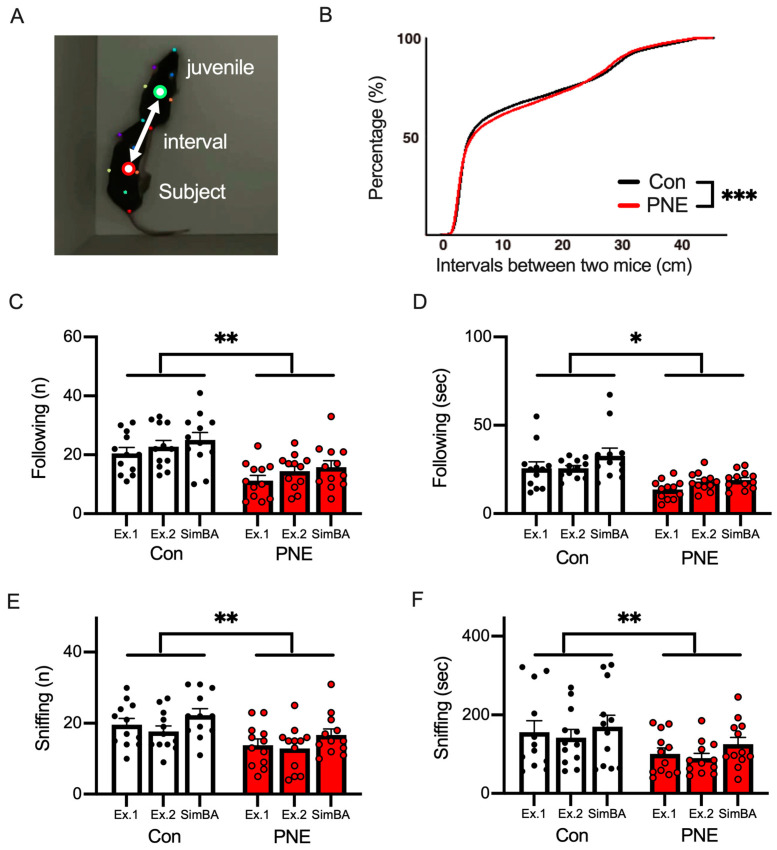
Machine-learning-based classification of social behaviors by SimBA revealed deficits caused by PNE in mice. (**A**): the interval between the juvenile and subject mice was defined as the distance between the centers of mass of the seven body parts of the juvenile (green circle) and subject (red circle) mice. (**B**): the accumulation curve of the intervals between the juvenile and Con/PNE subject mice. The distance between the juvenile and PNE subject mice was larger than in the case of the control subject mouse model (*** *p* < 0.001 by using K.S. test). (**C**): the number of following events is smaller in the PNE mouse model (*n* = 12) than in the control mouse model (*n* = 12). The same videos were analyzed by two human experimenters (Ex1 and Ex2) and by SimBA, a machine learning algorithm (ML) (t(2) = 30.47, ** *p* < 0.01). (**D**): the durations of time spent following by both the control and PNE mouse models are shown, and this was smaller in the PNE mouse model compared to the control mouse model (t(2) = 6.05, * *p* < 0.05). (**E**): the number of sniffing behaviors is shown, and fewer sniffing behaviors were observed in the case of the PNE mouse model (t(2) = 17.33, ** *p* < 0.01). (**F**): the duration of the sniffing behaviors was shortened in PNE mice (t(2) =15.70, ** *p* < 0.01).

To analyze the sociability of the PNE mice, we calculated the interval distance between the juvenile and subject mice (Figure 6A). We found that the interval distance during the juvenile interaction test was larger in PNE subject mice than in the control mice (Figure 6B). We further analyzed the social behaviors of following and sniffing, both of which were used to assess the sociability of the mouse (Supplementary Video S4 and Figure 6C–F). In this analysis, the frequency (Figure 6C,E) and the duration (Figure 6C,E) of following (Figure 6D,F) and sniffing (Figure 6F,G) behaviors were measured by two human experimenters (Ex1 and Ex2) and SimBA (ML). The frequency and the duration of following and sniffing were decreased in PNE mice in comparison with the control mice (Figure 6C–F). The correlations between the frequency and duration of these behaviors were high between human experimenters and the machine-learning-based analysis (Supplementary Figure S4). All these results indicate that sociability was affected by prenatal nicotine exposure in the mice.

### 3.6. Adult Hippocampal Neurogenesis in the Ventral Area of the Hippocampus Is Decreased in PNE Mice

**Figure 7:**
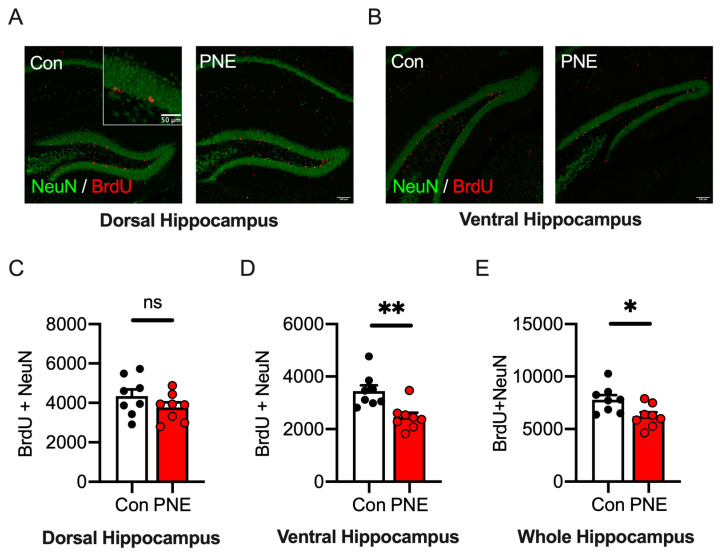
Newborn neurons in the ventral hippocampus were reduced via prenatal exposure to nicotine in mice. (**A**): representative pictures show histological images of newborn neurons in the dorsal hippocampus of the control (*n* = 8) and PNE (*n* = 8) mice. Newborn neurons are defined as cells positive for bromodeoxyuridine (BrdU, red) and a neuronal marker, NeuN (green). (**B**): pictures show the distribution of newborn neurons in the ventral hippocampus. Fewer BrdU/NeuN double positive cells were found in the ventral hippocampus of the PNE mouse model. (**C**): the number of newborn neurons in the dorsal hippocampus was not changed in the PNE mouse model compared to the control mouse model (t(14) = 1.33, *p* > 0.1). (**D**): the number of newborn neurons in the ventral hippocampus was significantly reduced in the PNE mouse model than in the control mouse model (t(14) = 3.53, *p* < 0.01). (**E**): the total number of newborn neurons in the entire hippocampus is shown and this was decreased in the PNE mouse model compared to the control mouse model (t(14) = 2.64, *p* < 0.01). Scale bar represents 100 μm (Figure 6A,B) and 50 μm (an inset of Figure 6A). ns = not statistically significant, * *p* < 0.05 ** *p* < 0.01, with Student’s *t*-test.

All these results supported the hypothesis that nicotine exposure during pregnancy is a risk factor for ASD, as well as for ADHD, in mice. Adult hippocampal neurogenesis is investigated as a hallmark of ASD in various mouse models [35,36,44]. To investigate adult neurogenesis in the hippocampus of PNE mice, we injected BrdU into 6-week-old mice and perfused the animals after two weeks; adult-born neurons were visualized by immunohistochemistry to BrdU and to a neuron marker, NeuN. The rodent hippocampus is known to be separated functionally into two subregions, the dorsal (septal, Figure 7A) and ventral (temporal, Figure 7B) subregions [45,46]. Therefore, we investigated adult neurogenesis in these two subregions of the hippocampus. The number of BrdU/NeuN double positive cells in the dorsal hippocampus was similar between the PNE and control mice (Figure 7C). On the other hand, the number of the BrdU/NeuN double positive cells in the ventral hippocampus was significantly decreased in PNE mice compared to the control mice (Figure 7D). We estimated the number of the adult-born neurons in the whole hippocampus and found that it was decreased significantly in PNE mice (Figure 7E). These data suggest that PNE causes a reduction in adult hippocampal neurogenesis in the ventral area of the hippocampus in mice.

## 4. Discussion

In this study, we optimized a set of parameters to establish a network in DeepLabCut in order to achieve accurate pose estimation. The combinational usage of the DLC-network and SimBA successfully detected the behavioral features of a single mouse and two mice of the same fur color, as precisely as if performed by human experimenters. We demonstrated that the PNE mice exhibited not only features of ADHD but also ASD behaviors, and that adult hippocampal neurogenesis in the ventral area of the hippocampus was attenuated in the PNE mouse model in comparison with the control mouse model.

Since smoking during pregnancy has been reported as a risk factor for ADHD in children [2], PNE rodents have been extensively studied in relation to ADHD. Behavioral assays of ADHD mouse models include the open-field test for activity level or the radial-arm or Y-maze tests for evaluating working memory. PNE mice have been shown to exhibit hyperactivity in the open-field test [20], but some papers failed to detect this hyperactivity [47]. A similar contradiction has been reported in terms of the working memory of PNE mice [48,49]. In this study, we did not observe an increase in the activity evaluated according to the distance traveled in the open-field test. In the same test, we also found increased anxiety in the PNE mice as indicated by the shorter time spent in the center. The travel distance and anxiety level interacted each other, which supports the results of our study (Figure 3). Therefore, the contradicting results regarding activity level in PNE mice may be due to the experimental environment or the handling methods, both of which could differ between research groups.

Impulsivity is a behavioral feature of ADHD in humans and has been examined in ADHD models including the PNE mouse model. We assessed impulsivity using the cliff avoidance reaction test and observed more frequent jumps from the platform and the shorter time elapses to the first jump [20,38]. The behavioral phenotype analyzed in the same method has been similarly observed in several laboratories using distinct models of ADHD. The consistency of behavioral outputs using the current protocol suggests that it is the most appropriate way to evaluate impulsivity in ADHD among the several protocols for the cliff avoidance test.

Another prominent phenotype of ADHD is impaired working memory. Working memory can be evaluated using several behavioral tests, such as the five-choice serial-reaction time test (5-CSRTT), the radial-arm maze, and the Y-maze. PNE rats have been subjected to 5-CSRTT and demonstrated an increased number of anticipatory responses and a decreased accuracy, indicating lower working memory [50]. In our study, we observed a decrease in spontaneous alteration without altering the total number of arm entries. The unchanged number of arm entries supported the notion that the PNE mice may not possess the ADHD feature of hyperactivity. Spontaneous alteration is considered an index to evaluate working memory in rodents. Therefore, the decrease in the spontaneous alteration in this study suggests that the working memory of the PNE mice was altered, which is another behavioral feature of ADHD. Taking all these results together, prenatal exposure to nicotine may cause ADHD in mice, as indicated by clinical reports in humans [2,6,8,9,12].

We assessed the social interaction of the PNE mice using the juvenile interaction test [51]. During the test, both mice were able to move freely in the arena. Free moving can reduce the psychological stress caused by physical restraint. The walking distance of the juvenile was not altered between the PNE and control mice, indicating that the presence of the PNE mice may not affect the behavior of the juvenile mice. However, the spatial interval between the juvenile and PNE mice was larger than that between the juvenile and control mice. Furthermore, contact behavior, such as the PNE mice following and sniffing the juvenile mice, decreased in the test. These results suggest that the PNE mice tend to keep a certain distance from other subjects and avoid social interaction. A decrease in child sociability after maternal smoking has been reported in some clinical reports [10,11,12]. The hypothesis that maternal smoking can be a risk factor for ASD is supported by our results using the mouse model in this study. Therefore, prenatal exposure to nicotine could become not only a risk factor for ADHD but also for comorbid ADHD and ASD.

Recent studies have suggested that the dopaminergic system is part of the biological mechanism of ADHD [52,53] and ASD [54,55]. The “dopamine hypothesis” is also supported by genetic and pharmacological studies in animal models [56,57,58]. It has been reported that dopamine release is reduced in the brain of PNE mice [20]. Animal studies using PNE mouse models have shown that methylphenidate, a dopamine reuptake inhibitor and a primary medication for ADHD, ameliorates neurophysiological and behavioral deficits [59]. To our knowledge, no dopamine-related compounds have been investigated to ameliorate social deficits in PNE mice. Some partial agonists of dopamine receptors, such as aripiprazole and cariprazine, have been reported to improve social behavior [60]. Future studies are needed to evaluate the efficacy of these treatments for ASD and ADHD phenotypes in PNE mice.

One of the aims of this study was the practical use of deep-learning-based analysis to evaluate the behavioral phenotypes of mice with similar appearances. After DeepLabCut became available, its labeling capabilities have been used in various studies, and the subsequent labeled data have been used to analyze interactions between multiple animals [61]. Labeling quality is crucial for behavioral analysis, and the labeling of multiple animals depends on the differences in the appearances of the individuals. In this study, we tested three video parameters for the training data and found the number of iterations improves the labeling quality of a single animal; we also found that the amount of training data is important in the case of two animals, suggesting that the labelling of single/multiple animals may require a different strategy for optimization. We would like to emphasize the importance of the number of animals for building a network. Especially in the case of multiple animals, we found a correlation between the number of videos (animals) and the quality of the estimation (Figure 5B), indicating that a variety of training data is necessary to construct a network that accurately estimates body parts. In addition, increasing the number of animals tested allows for a robust analysis of animal behavior. Taken together, these results indicate that the number of animals is an important factor in building a network and obtaining high-quality results. We found a high correlation between the behavior detection performance of SimBA and human experimenters. In the experiments using a single animal, a video from the top view enabled SimBA to detect three-dimensional (3D) behaviors such as grooming and rearing. Recently, 3D pose estimation became available in the DeepLabCut implemented software [27,62], which will allow us to analyze these behaviors with high precision.

Neurogenesis occurs throughout life in two different brain regions, the subventricular zone of the lateral ventricle and the dentate gyrus of the hippocampus. Hippocampal neurogenesis has been shown to be associated with neuropsychiatric disorders such as anxiety, depression, schizophrenia, and ASD. Studies have demonstrated that adult neurogenesis in the hippocampus is impaired in various animal models of ASD, such as Shank3 knockout, Neuroligin3^R451C^ point-mutant, prenatal valproic-acid exposure, and BTBR mice [35,44,63,64]. We found a decrease in newborn neurons in the hippocampus, specifically in the ventral part. The ventral hippocampus is associated with emotional states such as anxiety and fear. In this study, we observed that the PNE mice showed increased anxiety in the open-field test, which is in line with the idea that anxiety levels are elevated in ASD. Recently, studies have indicated that the ventral hippocampus as an essential brain region of ASD behavioral phenotypes, which are closely correlated to anxiety level. Therefore, the specific down-regulation of BrdU-positive neurons in the ventral hippocampus may be involved in the neuronal mechanism underlying lowered social behaviors in ASD.

Recent neuroimaging studies have suggested differences between pure ADHD and comorbid ADHD and ASD [65] and these two groups may need distinct treatment [66]. Future studies investigating the behavioral phenotypes of PNE humans and animals will help us to establish a more efficient treatment for comorbid ADHD and ASD in PNE children.

## 5. Conclusions

In this study, we analyzed the behavioral features of PNE mice using deep-learning-based algorithms such as DeepLabCut and SimBA. The machine learning methods provided a blind and unbiased analysis of the animal behaviors and revealed that PNE mice shared behavioral phenotypes featuring in ADHD. In addition to ADHD-associated features, we also found a deficit in social interaction between a juvenile mouse and a PNE mouse model. A decreased number of newborn neurons in the hippocampus, which is a hallmark of ASD pathology, was observed in the PNE mouse model. All these results suggest that prenatal exposure to nicotine may induce comorbid ADHD and ASD, rather than ADHD alone.

## Data Availability

All datasets used and/or analyzed during this current study are available from the corresponding authors on reasonable request.

## Supplementary Materials

The following supporting information can be downloaded at https://www.mdpi.com/article/10.3390/cells13030275/s1, Figure S1: Evaluation of labeling body-parts on a single animal by different conditions of training. RMSE from train data (black bars) and test data (red bars) is indicated. Parameters (the numbers of videos, frames, and iterations) are described on *X* axis; Figure S2: Correlation analysis of event-detection performance between SimBA (Machine learning based algorism) and human experimenters. The number or the duration of grooming and rearing behaviors detected by SimBA (*X*-axis), and human experimenters (*Y*-axis) were plotted. Measurements by two human experimenters were represented by blue (Ex1) or orange (Ex2) makers; Figure S3: Evaluation of labeling body-parts on two C57BL/6J mice by different conditions of training. RMSE from train data (black bars) and test data (red bars) is indicated. Parameters (the numbers of videos, frames, and iterations) are described on *X* axis; Figure S4: Correlation analysis of event-detection performance between SimBA (Machine learning based algorism) and human experimenters. The number or the duration of social interactions, following and sniffing, detected by SimBA (*X*-axis) and human experimenters (*Y*-axis) were plotted. Measurements by two human experimenters were represented by blue (Ex1) or orange (Ex2) makers; Video S1: Pose estimation of a single mouse during the open field test by DeepLabCut; Video S2: Behavioral annotation by SimBA; Video S3: Pose estimation of two mice by DepLabCut; Video S4:Behavioral annotation of social interaction by SimBA.
