# Supplementary material for: Deep-Learning-Based Analysis Reveals a Social Behavior Deficit in Mice Exposed Prenatally to Nicotine
Source: Cells. 2024 Feb 1;13(3):275. doi: 10.3390/cells13030275 (PMC10855062; doi:10.3390/cells13030275)
Supplement: Supplementary file 1 [file cells-13-00275-s001.zip › Legends for supplementary figures.pdf]

Legends for supplementary figures

Supplementary Figure S1

Evaluation of labeling body-parts on a single animal by different conditions of training. RMSE from train data (black bars) and test data (red bars) is indicated. Parameters (the numbers of videos, frames, and iterations) are described on X axis.

Supplementary Figure S2

Correlation analysis of event-detection performance between SimBA (Machine learning based algorism) and human experimenters. The number or the duration of grooming and rearing behaviors detected by SimBA (X-axis), and human experimenters (Y-axis) were plotted. Measurements by two human experimenters were represented by blue (Ex1) or orange (Ex2) makers.

Supplementary Figure S3

Evaluation of labeling body-parts on two C57BL/6J mice by different conditions of training. RMSE from train data (black bars) and test data (red bars) is indicated. Parameters (the numbers of videos, frames, and iterations) are described on X axis.

Supplementary Figure S4

Correlation analysis of event-detection performance between SimBA (Machine learning based algorism) and human experimenters. The number or the duration of social interactions, following and sniffing, detected by SimBA (X-axis) and human experimenters (Y-axis) were plotted. Measurements by two human experimenters were represented by blue (Ex1) or orange (Ex2) makers.
