# Supplementary material for: Deep-Learning-Based Analysis Reveals a Social Behavior Deficit in Mice Exposed Prenatally to Nicotine
Source: Cells. 2024 Feb 1;13(3):275. doi: 10.3390/cells13030275 (PMC10855062; doi:10.3390/cells13030275)
Supplement: Supplementary file 1 [file cells-13-00275-s001.zip › SupplementaryFigures.pdf]

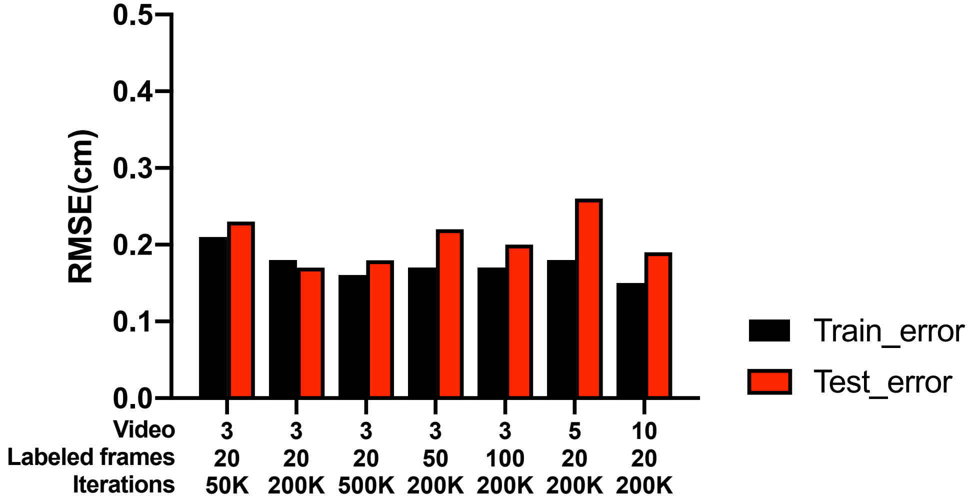
0.5
0.4
0.3
0.2
0.1
0.0
RMSE(cm)
Train_error
Test_error
Video
Labeled frames
Iterations
3
20
50K
3
20
200K
3
20
500K
3
50
200K
3
100
200K
5
20
200K
10
20
200K

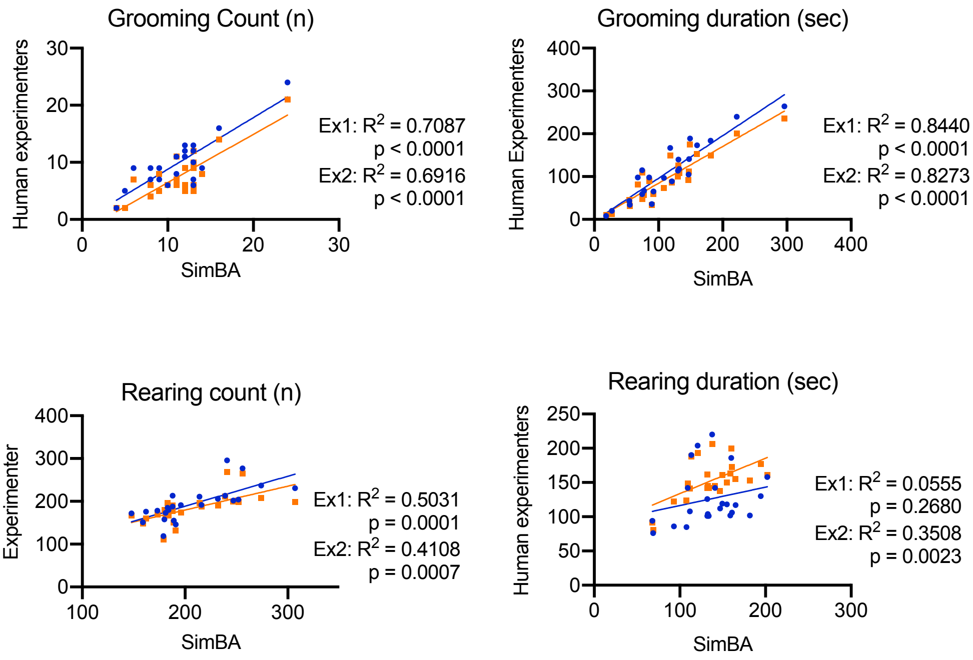
Grooming Count (n)
Human experimenters
SimBA
Ex1: R2 = 0.7087
p < 0.0001
Ex2: R2 = 0.6916
p < 0.0001
Grooming duration (sec)
Human Experimenters
SimBA
Ex1: R2 = 0.8440
p < 0.0001
Ex2: R2 = 0.8273
p < 0.0001
Rearing count (n)
Experimenter
SimBA
Ex1: R2 = 0.5031
p = 0.0001
Ex2: R2 = 0.4108
p = 0.0007
Rearing duration (sec)
Human experimenters
SimBA
Ex1: R2 = 0.0555
p = 0.2680
Ex2: R2 = 0.3508
p = 0.0023

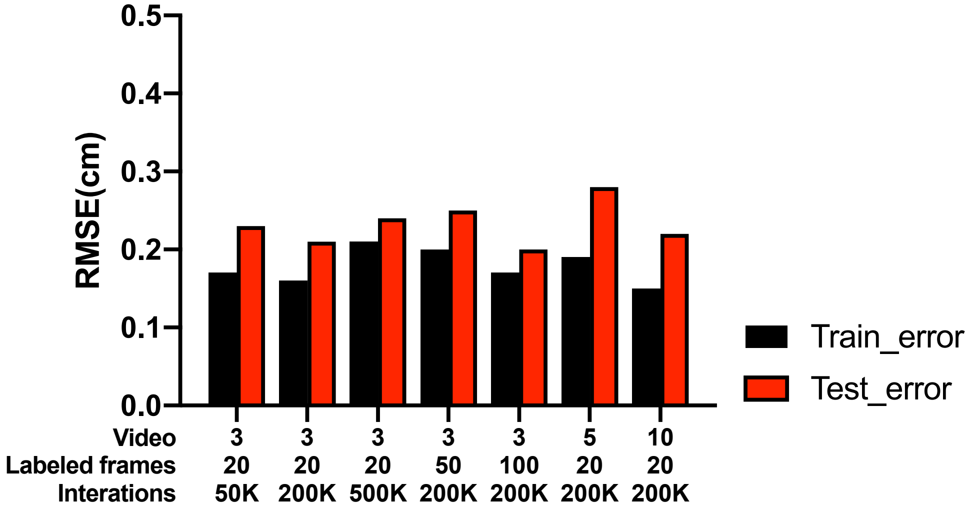

RMSE(cm)
0.5
0.4
0.3
0.2
0.1
0.0
Video
Labeled frames
Interations
3
20
50K
3
20
200K
3
20
500K
3
50
200K
3
100
200K
5
20
200K
10
20
200K
Train_error
Test_error

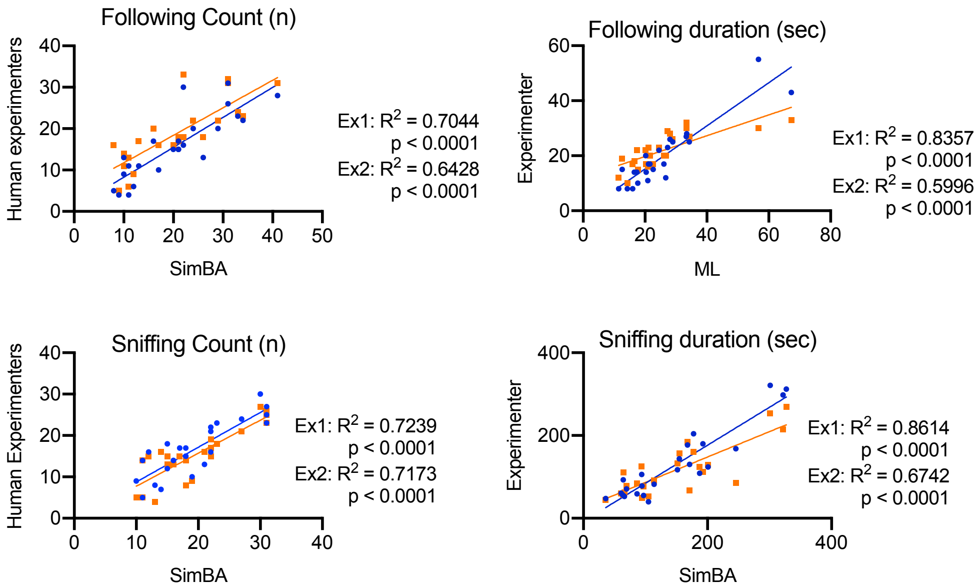
Following Count (n)
Human experimenters
SimBA
Ex1: R2 = 0.7044
p < 0.0001
Ex2: R2 = 0.6428
p < 0.0001
Following duration (sec)
Experimenter
ML
Ex1: R2 = 0.8357
p < 0.0001
Ex2: R2 = 0.5996
p < 0.0001
Sniffing Count (n)
Human Experimenters
SimBA
Ex1: R2 = 0.7239
p < 0.0001
Ex2: R2 = 0.7173
p < 0.0001
Sniffing duration (sec)
Experimenter
SimBA
Ex1: R2 = 0.8614
p < 0.0001
Ex2: R2 = 0.6742
p < 0.0001
